# Reliability of movement workspace measurements in a passive arm orthosis used in spinal cord injury rehabilitation

**DOI:** 10.1186/1743-0003-9-37

**Published:** 2012-06-09

**Authors:** Claudia Rudhe, Urs Albisser, Michelle L Starkey, Armin Curt, Marc Bolliger

**Affiliations:** 1 Spinal Cord Injury Center, Balgrist University Hospital, Forchstrasse, 340, 8008, Zurich, Switzerland

**Keywords:** Passive arm orthosis, Spinal cord injury, Upper limb function, Rehabilitation, Reliability

## Abstract

**Background:**

Robotic and non-robotic training devices are increasingly being used in the rehabilitation of upper limb function in subjects with neurological disorders. As well as being used for training such devices can also provide ongoing assessments during the training sessions. Therefore, it is mandatory to understand the reliability and validity of such measurements when used in a clinical setting. The aim of this study was to evaluate the reliability of movement measures as assessed in the Armeo Spring system for the eventual application to the rehabilitation of patients suffering from cervical spinal cord injury (SCI).

**Methods:**

Reliability (intra- and inter-rater reliability) of the movement workspace (representing multiple ranges of movement) and the influence of varying seating conditions (5 different chair conditions) was assessed in twenty control subjects. In eight patients with cervical SCI the test-retest reliability (tested twice on the same day by the same rater) was assessed as well as a correlation of the movement workspace to retrieve self-care items as scored by the spinal cord independence measure (SCIM 3).

**Results:**

Analysis of workspace measures in control subjects revealed intra-class correlation coefficients (ICC) ranging from 0.747 to 0.837 for the intra-rater reliability and from 0.661 to 0.855 for the inter-rater reliability. Test-retest analysis in SCI patients showed a similar high reliability with ICC = 0.858. Also the reliability of the movement workspace between different seating conditions was good with ICCs ranging from 0.844 to 0.915. The movement workspace correlated significantly with the SCIM3 self-care items (p < 0.05, rho = 0.72).

**Conclusion:**

The upper limb movement workspace measures assessed in the Armeo Spring device revealed fair to good clinical reliability. These findings suggest that measures retrieved from such a training device can be used to monitor changes in upper limb function over time. The correlation between the workspace measures and SCIM3 self-care items indicates that such measures might also be valuable to document the progress of clinical rehabilitation, however further detailed studies are required.

## Background

Over the last decades, many robotic devices have been developed for upper extremity rehabilitation after neurological disorders, for example, current established systems include the MIT-Manus [1], the Assisted Rehabilitation and Measurement (ARM)Guide [2], the Mirror Image Motion Enabler (MIME) [3], the Bi-Manu-Track [4] and the ARMin [5]. Although the design and development of all these robotic devices have been extensively reported only a few studies were performed as part of a regular rehabilitation program and mainly focused on the effectiveness of specific training sessions or specific patient groups [6-8]. The main goal of these devices is to increase the intensity and quality of rehabilitation therapy [9] by providing well-controlled and highly repeatable conditions as well as optimized assistance to the patient [10,11]. In addition these devices are able to reduce the work load of the therapist by assisting specific movements of the patients and supporting the weight of the patients arm during therapy [12].

In the field of SCI rehabilitation passive arm orthoses are receiving increased interest, such as the Therapy Wilmington Robotic Exoskeleton (T-WREX) [13-15] and its modified and commercialized version, the Armeo Spring (Hocoma AG, Volketswil, Switzerland). These non-robotic, gravity support systems are based on an ergonomic arm exoskeleton with integrated springs. Such devices cradle the entire arm, from shoulder to the hand, and counterbalance the weight of the patients’ arm. They enhance any residual function and neuromuscular control and assist active movement across a large 3-D workspace providing an augmented feedback [16]. As there are no actuators implemented in these devices, all movements are generated by the users themselves.

The passive orthoses and robotic devices are equipped with sensors responsible for the assessment of their multiple degrees of freedom as well as to display the movement of different joints. Therefore, enormous amounts of data are collected during training that could be used not only to monitor the training session (intensity, duration, frequency etc.) but also to follow changes in the functional impairment. Recently studies have started to focus on the effectiveness of training with a gravity compensation device in different patient groups [16-18]. However, psychometric properties (reliability and validation) that account for clinical and patient-relevant aspects (such as the influence of the positioning of the patient) have not been sufficiently addressed.

The Armeo Spring system is frequently used in the rehabilitation of upper limb function in stroke as well as cervical spinal cord injured patients. The device has seven degrees of freedom and is equipped with seven potentiometers (resolution: 0.2°) to measure the joints angles. These measurements are used to calculate the endpoint position of the hand in space. In addition, one pressure sensor is placed in the handle to assess closing and opening of the hand.

In patients with cervical spinal cord injuries major physical changes have been reported to occur during rehabilitation [19] such as improvements in the seating conditions (from electric and reclining wheelchairs to eventual use of regular chairs), trunk stability and limb function. These patient conditions and also the construction of wheelchairs (often bulky, electrical wheelchairs) impose constraints on the placement of the patient within the device. Therefore, assessments obtained during gravity support system training might be influenced by these imposed constraints resulting in unknown effects on the retrieved measures. Furthermore, it needs to be established how valuable these assessments are for clinical documentation and monitoring of functional changes during rehabilitation. Therefore, the aim of this study was to evaluate (1) the reliability of the movement measurements (i.e. workspace) in controls and patients with cervical SCI, (2) the influence of 5 different seating conditions on measures of movement, and (3) the correlation between the movement workspace in cervical SCI and functional abilities in daily life.

## Method

### Movement workspace measurement with the ARMEO Spring

**Figure 1:**
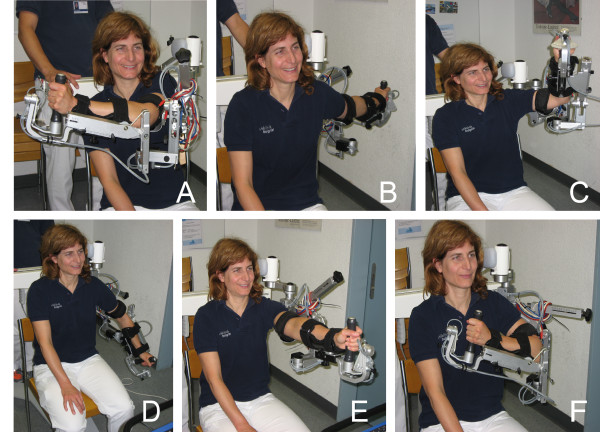
**Tested arm movements and positions.** Maximal reaching of a healthy subject to the A: right; B: left; C: top; D: bottom; E: far and F: close.

To measure a subject’s movement workspace in the ARMEO Spring**,** the subject has to be seated on a chair. The device is then aligned to the patient. The alignment reference of the device to the subject is the vertical axis through the subject’s shoulder joint (humero-scapular joint). The subject’s arm is fitted to the exoskeleton and the height of the device as well as the upper and lower arm length and upper and lower arm weight support are defined individually for each subject, according to the user instructions. The movement workspace was calculated by using the x (right-left movement), y (up-down movement) and z (far-close movement) axes of the Cartesian coordinate system with its origin set as the shoulder joint of the device. Subjects are asked to move their arm to the maximal right position, whilst maintaining a straight and stable trunk position, and to hold this position for 3–5 seconds, then move the arm to the maximal left position keeping a stable position and again holding the position for 3–5 seconds, and so forth for maximal top, bottom, forward and close (hand in front of the chest) position (see Figure 1*).* Subjects were not provided with knowledge about their results. During the movement the positions of the endpoint (hand) were recorded using the standard ARMEO Spring software [9].

### Participants

The study protocol was approved by the local Ethics committee and conformed to the Declaration of Helsinki. All participants were able to understand and follow the instructions and gave written informed consent before data collection.

Twenty subjects without neurological deficits (mean age 35 years, SD 11 years; 15 women and 5 men) participated in the study. Additionally 8 subjects with defined neurological deficits in the upper extremity (mean age 49.6 years, SD 12.4 years; 4 women and 4 men) participated in the study. Characteristics of the subjects with neurological deficits in the upper extremity are shown in Table 1.

**Table 1:** Characteristics of subjects with neurological deficits in the upper extremity

Subject No.	Sex	Age (years)	Type of injury	Time since injury (month)	SCIM sub score self-care	Seating	Tested Arm
S1	M	67	Cervical SCI (C4 ASIA D)	3	9	Electric w/c	L + R
S2	M	47	Guillain-Barré Syndrome	12	2	Electric w/c	L + R
S3	M	40	Tetraplegia after with brain stem lesion	6	0	Electric w/c	L + R
S4	M	63	Cervical SCI (C4 ASIA D)	29	9	Regular chair	L + R
S5	F	40	Guillain-Barré Syndrome	2	19	Regular chair	L + R
S6	F	35	Cervical SCI (C4 ASIA C)	19	0	Electric w/c	L + R
S7	F	43	Guillain-Barré Syndrome	2	4	Manual w/c	L + R
S8	F	62	Cervical SCI (C3 ASIA C)	4	0	Electric w/c	L + R

Abbreviations: SCI: Spinal Cord Injury; ASIA: American Spinal Cord Injury Association – standard neurological classification; w/c: wheelchair

### Study protocols

**Figure 2:**
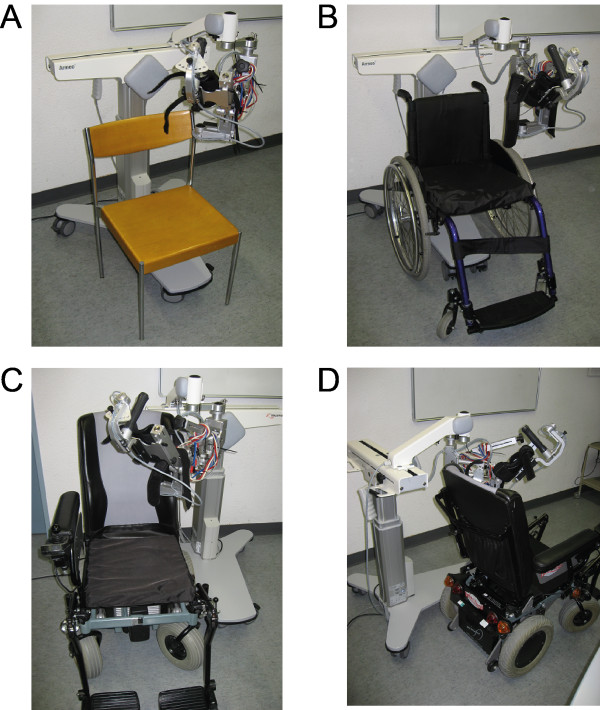
**Seating used for movement workspace reliability evaluation between different seating.** A: regular wooden chair; B: manual wheelchair used for two conditions: sitting straight, sitting in hip forward position; C: electric wheelchair and D: electric wheelchair with device positioned with a deviation angle of 10° to the wheelchair axe.

In the control subjects three different tests were performed:

1) Reliability of the movement workspace between 5 different seating conditions: Two sessions were performed within 7–10 days. Session one was a training session to familiarize the subject with the procedure. Data from session two was used for analysis. One session comprised 5 measurements. For each measurement a different seating condition was used a) straight sitting in a regular chair (wooden seat) with low seat and back support (rc); b) straight sitting in a lightweight manual wheelchair (w/c); c) “relaxed” sitting position (hip forward position with flexion of the trunk) in the same lightweight manual wheelchair (w/c f); d) straight sitting in an electric wheelchair (e w/c); e) straight sitting in an electric wheelchair with the device placed in an deviation angle of 10° to the horizontal axis of the wheelchair (A dev) (Figure 2). Measurements were taken of the left arm only. The subject’s upper and lower arm length and upper and lower arm weight support were defined during the first session and used for all subsequent tests.

2) Intra-rater reliability of the movement workspace was calculated for all seating conditions: the subjects left arms were tested twice by the same rater within 7–10 days.

3) Inter-rater reliability of the movement workspace was calculated for all seating conditions: the subjects left arms were tested twice within 7–10days first by rater A and second by rater B. Both raters were experienced in the use of this device and donning and doffing it to patients/subjects. Each rater was blinded to the results obtained by the other rater.

In the patients with upper limb impairment the following test was performed:

1) Test-retest reliability of the movement workspace: both arms of the subjects with neurological deficits were measured twice within one day by the same rater. Subjects used whatever chair they used at their current stage of rehabilitation as seating for the measurements. Both arms were tested alternately. This assured a break for each arm prior to the measurement of approximately 10 minutes in addition to the time taken to apply the device to the arm. Arm length and weight support were defined for each side separately before the first measurement and were re-used for the second measurement. Positioning and height were readjusted before each measurement.

2) To assess functional ability in daily life, the Spinal Cord Independence Measure 3 (SCIM3) [20,21] was used. The SCIM3 is a standard clinical assessment tool to measure independence in daily activities in subjects suffering from a spinal cord injury. The SCIM3 was administered within one week of the measurement in the Armeo device. The sub total score of the self-care items of the SCIM3 was used for correlation with the volumes of the movement workspace.

### Data analysis

Data on the angular position were recorded at a sampling rate of 50 Hz and stored on a standard PC. At the completion of each test, the maximal angular displacement in a 4000 ms interval after the start cue was calculated using a moving average (width 2000 ms).

To evaluate reliability between different seating conditions as well as intra- and inter-rater reliability, the volume of the active movement workspace [22,23] was calculated for each measurement. Therefore, distances between the endpoints of each movement axis (left-right, top-bottom, far-close) were calculated and then multiplied. The volume is displayed in m^3^.

Reliability was evaluated using analysis of variance (ANOVA)-based intraclass correlation coefficients (ICC). ICC scores were compared with the following scale for interpretation of correlation: good (1.00-0.8), fair (0.80-0.60), and poor (< 0.60) [24]. ICC > 0.80 has been suggested to be feasible for clinical work but also ICC between 0.60 and 0.80 can provide researchers with valuable information [24].

To calculate the correlation between the movement workspace and the SCIM3 self-care sub score, the movement workspace data were normalised according to subjects arm length. As the movement workspace assesses both arms independently whereas the SCIM3 does not differentiate between sides, an average of the normalised movement workspace between the right and the left arms of the two measurements was used. Correlations were calculated using the Spearman rank correlation.

All statistics were calculated with SPSS (SPSS 17 for Windows, SPSS Inc., Chicago, IL, USA).

## Results

ICC between seating conditions showed good reliability between all conditions with ICC between 0.844 and 0.915 (Table 2).

**Table 2:** Reliability between different seating (ICC)

Seating (N = 20)	ICC	CV_ME_
rc - w/c	0.897	33.6
rc – w/c f	0.868	32.6
rc – E w/c	0.893	34.7
rc – A dev	0.844	32.6
w/c – w/c f	0.87	32.6
w/c – E w/c	0.883	31.7
w/c – A dev	0.852	34.7
w/c f – E w/c	0.915	31.7
w/c f – A dev	0.865	34.6
E w/c – A dev	0.868	34.6

Abbreviations: rc: regular chair; w/c: wheelchair; w/c f: wheelchair forward position; E w/c: electric wheelchair; A dev: ARMEO Spring deviated position; ICC: Intraclass correlation coefficient; CV_ME_: coefficient of variation of the method error

Intra-rater reliability showed fair to good ICC ranking from 0.747 in the electric wheelchair to 0.837 in the manual wheelchair. The coefficient of variation (CV_ME_) was between 33.5-35.6% (Table 3). Only 18 subjects could be measured in the condition ‘ARMEO Spring deviated’ due to technical reasons (N = 18). In all other conditions all 20 subjects were measured (N = 20).

**Table 3:** **Intra-rater and inter-rater reliability for subjects without neurological deficits (ICC, CV**_**ME**_**)**

Seating (N = 20)	Intra-rater		Inter-rater	
	ICC	CV_ME_	ICC	CV_ME_
rc	0.787	34.2	0.852	33.5
w/c	0.837	34.5	0.791	32.6
w/c f	0.764	33.9	0.855	32.6
E w/c	0.747	33.4	0.837	31.3
A dev	0.795 (N = 18)	35.6	0.661 (N = 16)	33.0

Abbreviations: rc: regular chair; w/c: wheelchair; w/c f: wheelchair forward position; E w/c: electric wheelchair; A dev: ARMEO Spring deviated position; ICC: Intraclass correlation coefficient; CV_ME_: coefficient of variation of the method error

Inter-rater reliability showed fair to good reliability with ICC from 0.661 in the condition ‘ARMEO Spring deviated’ to 0.855 in ‘manual wheelchair forward position’. Also for the inter-rater reliability the CV_ME_ was between 31.3-33.5% (Table 3). Only 16 subjects could be measured for the condition ‘ARMEO Spring deviated’ due to technical reasons (N = 16). In all other conditions all 20 subjects were tested (N = 20).

In the patients with upper limb impairment the following test was performed: Two subjects with neurological deficits in the upper extremity were too weak in one arm to perform any measurable voluntary movement. Therefore there were a total of 14 measurements for the test-retest reliability in subjects with neurological deficits. Results revealed a good reliability with ICC = 0.858 and a CV_ME_ = 34.1% (N = 14).

The average normalised movement workspace volume between the right and left arm correlated significantly with the SCIM3 sub score self-care (rho = 0.72, p < 0.05).

## Discussion

The present study in controls and a limited number of patients showed that measurements of upper limb movements taken by the Armeo Spring device are reliable and feasible in a clinical setting. The psychometric properties addressing re-testing and the influence of seating conditions were very favourable supporting the eventual introduction of such measures into clinical protocols.

### Psychometric properties

The Armeo Spring device allowed to record reliable data regarding the movement workspace when tested under differing conditions as well as by different testers. Even in conditions where patients changed their seating device (which is a natural condition in patients recovering from SCI) the reliability of the movement workspace data was not affected. It has to be noted though that the coefficient of variation of the method (CV_ME_) error is relatively high in all cases with 31.3-35.6%. Changes of the workspace below CV_ME_ cannot be attributed to a performance change and must be handled as trial-to-trial noise. Although reliability between different seating conditions appears to be good a seating condition providing a good hip and back support is recommended. This is due to the observation that compensatory and trunk movements occurred more often on the regular chair and needed verbal prompting for correction. The increased instability is most likely due to a lower support and guidance of the body from a regular chair, compared to all other used clinical seating conditions. This conclusion is supported by Aissaoui et al. [25] describing the effects of different seat cushions on the reaching ability of paralysed subjects. They showed that the dynamic stability in sitting was an important factor on the reaching ability of the subjects. May et al. [26] showed that a special back-support (J2 back) on the wheelchair which provides additional support and stability significantly improved the forward reaching function of 27 subjects with SCI compared to the normal wheelchair back rest.

Although data did not show weaker results for the condition ‘ARMEO Spring deviated’ the importance of a precise alignment of the subject and the device has to be pointed out due to clinical reasons. Patients experience more restriction in the movement possibilities due to the occurrence of joint limitations on the exoskeleton and thus may feel discomfort if the alignment is not optimal. This is an important point as the correct alignment of the device and the subject is often a problem in clinical practice. Electric wheelchairs, breathing aids, special arm supports or reclining chairs are common, especially in the early stages of rehabilitation. In this early stage, the use of the gravity support system would be most beneficial, as other training techniques are often limited because of the patient’s general condition.

### Assessment of movement workspace

Although the used movement workspace in the tested device has the shape of a cube instead of the anatomical spherical shape the findings were found to be related to clinical outcomes. Klopčar et al. [22] and Robinson et al. [23] describe the clinical relevance of the movement workspace when assessing shoulder function. Klopčar et al. documented the rehabilitation progress of a subject with a frozen shoulder with a 3D arm-reachable workspace [22]. Robinson objectively quantified a three-dimensional reachable workspace of subjects with tetraplegia using an eight camera opto-electronic system [23]. The workspace volume can be easily calculated from the data provided by the Armeo device and be followed over time to document changes during the course of rehabilitation.

The movement workspace is a multiple joint measure and does not assess the maximal shoulder movement capacity in a single direction as assessed in a single joint range-of-motion measurement. However, the reliability information from this more functional movement seems to be very good compared to, for example, single joint goniometry measurements of the shoulder. Reliability studies for goniometry measurements in the shoulder have a large intra- and inter-rater variability in results. Hayes et al. [27] tested 17 subjects with shoulder pathology with different methods. The inter-rater reliability for the shoulder goniometry was ICC = 0.64-0.69 and for the intra-rater reliability ICC = 0.53-0.65. Better results were found in healthy subjects which had an ICC = 0.83-0.96 (inter-rater) and ICC = 0.74-0.94 (intra-rater) [28] and in a group with subjects with and without shoulder pathology, who had an ICC = 0.36-0.91 (inter-rater) and ICC = 0.76-0.94 (intra-rater) [29]. In these studies the reliability of goniometry measurements was also largely dependent on the specific movement direction.

### Clinical appreciation of movement workspace

The Armeo movement workspace was significantly correlated with the SCIM sub score for the self-care items. It was shown previously that the SCIM sub score reliably assesses function of the upper extremity and is able to document changes over the course of rehabilitation [30] and appears to be an appropriate measure for the validation of other measures of upper extremity function in SCI subjects when referring to activities in daily life.

In a first step we performed data-analysis of the movement average of 2000 ms of each reaching direction as well as the maximal scores (maximal reaching endpoint for each direction). The results of these analyses showed the importance of holding the end position of a movement for a few seconds in order to obtain reliable results as subjects with neurological deficits were able to reach further when using the continuing swing of a movement. However, this swing could not be controlled voluntarily and therefore the arm could not be stabilized in this position due to a lack of muscular control in the distal arm. In an everyday situation, the swing of a movement might be helpful, e.g., when using a light switch, where a short touch is sufficient to press the bottom. Although in most other activities it is crucial to be able to stabilise the hand and arm in a certain position in order to grasp or manipulate objects. Therefore, we concluded that using the maximal scores for volume calculation is not suitable but instead the average score of two seconds holding the end position can be assumed to produces representative results for a subject’s reachable workspace. This indirect finding might be important with respect to the design of new assessments for the device as stability in holding a position seems to be crucial for assessing the maximal reaching capacity in a subject.

This study had a limited number of subjects. To fully evaluate the reliability and validity of the assessment capacity of this device, further studies will need to be performed, with a larger number of SCI patients and also patients with different neurological deficits, e.g., different levels and completeness of SCI, stroke, multiple sclerosis. To be able to perform these analyses with the requested number of patients within a reasonable amount of time we aim to perform a multicentre study.

## Conclusions

Measures of the movement workspace of the upper limb as provided by the Armeo Spring are reliable for clinical use. They have been shown to be less affected by changes in seating conditions and measurements by different examiners are reliable. The correlation of the movement workspace to measures of functional impairment is favourable for recording the effects of training over time and for the estimation of the clinical course of recovery. Based on these preliminary findings further studies with larger sample sizes of patients with different levels and completeness of spinal cord injury are warranted to develop training and assessment protocols using the Armeo Spring.

## Abbreviations

SCIM3, Spinal Cord Independence Measure 3; SCI, Spinal cord injury; ASIA, American Spinal Cord Injury Association –standard neurological classification; rc, regular chair; w/c, wheelchair; w/c f, wheelchair forward position; e w/c, electric wheelchair; A dev, Armeo Spring deviated position; ICC, Intraclass correlation coefficient; CVME, coefficient of variation of the method error.

## Competing interests

All authors are employed by the Spinal Cord Injury Center of the University Hospital Balgrist. AC is Director of the Spinal Cord Injury Center of the University Hospital Balgrist and Professor for Paraplegiology at the University of Zurich, Switzerland.

## Authors’ contributions

CR developed the study design, performed data acquisition, completed data analysis and wrote the manuscript. UA aided in the study design, in the data acquisition and in revising the manuscript. MS provided expert guidance on experimental design and edited the manuscript. AC provided expert guidance on experimental design and edited the manuscript. MB aided in the study design and with data analysis as well as in revising the manuscript. All authors read and approved the final manuscript.
